# Phylogeographic Characterizations of Recent (2015–2023) Senecavirus A Isolates from Canada

**DOI:** 10.3390/v17020141

**Published:** 2025-01-22

**Authors:** Kate Hole, Oksana Vernygora, Katherine Handel, Michelle Nebroski, Oliver Lung, Charles Nfon, Shawn Babiuk

**Affiliations:** 1 National Centre for Foreign Animal Disease, Winnipeg, MB R3E 3M4, Canada; oksana.vernygora@inspection.gc.ca (O.V.); katherine.handel@inspection.gc.ca (K.H.); michelle.nebroski@inspection.gc.ca (M.N.); oliver.lung@inspection.gc.ca (O.L.); charles.nfon@inspection.gc.ca (C.N.); 2 Department of Biological Sciences, University of Manitoba, Winnipeg, MB R3T 2N2, Canada; 3 Department of Immunology, University of Manitoba, Winnipeg, MB R3E 0T5, Canada

**Keywords:** Senecavirus A, phylogeographic analysis, recombination

## Abstract

Senecavirus A (SVA) continues to cause vesicular lesions in swine in Canada and many regions worldwide. Since the vesicular lesions caused by SVA are similar to those caused by foot and mouth disease virus, swine vesicular disease virus and vesicular stomatitis virus, a foreign animal disease investigation must be initiated to rule out these diseases. SVA isolates from pigs displaying vesicular lesions in Canada from 2015 to 2023 were sequenced, and phylogeographic analysis was performed using the complete genome sequences. The results infer that SVA has spread between the United States and Canada several times. In addition, the results suggest that SVA spreads from different regions. SVA spread was inferred from Canada into Thailand, India and Mexico and inferred from the United States to Brazil, Columbia, Chile and China with ten separate introductions. Furthermore, recombination was observed in SVA genomes from Canada, the United States and China.

## 1. Introduction

Senecavirus A (SVA), formerly Seneca Valley Virus, belongs to the family *Picornaviridae*. Picornaviruses are non-enveloped, single-stranded, positive sense RNA viruses, which include 63 genera containing 147 species, with many additional viruses awaiting classification [1]. The most important picornaviruses affecting agriculture are *Aphthovirus*, which includes the species foot-and-mouth disease virus (FMDV) and *Enterovirus*, which includes the species swine vesicular disease virus (SVDV). SVA was first isolated as a cell culture contaminant in 2002 in the United States, and the first genome sequence of SVA was published in 2008 [2]. Several picorna-like viruses isolated from pigs with various clinical diseases in the United States between 1988 and 2005 were partially sequenced and found to show high similarity to SVA [3].

SVA causes a clinical disease characterized by vesicular lesions in pigs in the field and after experimental pig infections [4]. The minimum infectious dose of SVA was a Ct of 25.6 and a Ct of 29.7 for finishing pigs and neonates, respectively [5]. The first recorded occurrence of SVA in Canada in 2007 was when pigs exported to the United States presented with vesicular lesions similar to foot-and-mouth disease (FMD) [6]. Since 2015, an increased incidence of SVA has occurred worldwide. SVA has now been detected in multiple countries, including China, Brazil, Colombia, Thailand, India, Vietnam, Chile, Mexico and, most recently, England [6,7,8,9,10,11,12,13]. Most SVA infections are inapparent or cause very mild disease, illustrated by the low number of SVA disease investigations in Canada and the sequencing of SVA from an asymptomatic pig in China [14]. In addition, seroprevalence studies performed in the United States indicated that exposure to SVA ranges from 17.3% and 7.4% in breeding and growing pig farms, respectively [15]. SVA can likely spread without clinical disease characterized by vesicular lesions and is only noticed when there are vesicular lesions. It has recently been demonstrated that sows persistently infected with SVA can transmit to contact piglets [15]. In addition, stress can increase the disease severity of SVA [15]. In 2015, SVA clinical disease occurred on a much larger scale in Canada, demonstrating that under certain circumstances, including stress and movement of swine to assembly yards, SVA can spread and cause clinical disease in pigs [16].

Unlike FMD, SVA is not a reportable disease to the World Organization for Animal Health (WOAH)/Food and Agriculture Organization of the United Nations (FAO). However, its presence does trigger foreign animal disease (FAD) investigations in Canada and other countries free of FMD due to the similarity of its clinical presentation to foot and mouth disease, swine vesicular disease (SVD) and vesicular stomatitis (VS). SVA is diagnosed using real-time reverse transcription polymerase chain reaction (RRT-PCR) and virus isolation, and there is a competitive ELISA available to assess antibody responses in pigs [17]. Since SVA is not a reportable disease, the understanding of the epidemiology of SVA is limited. SVA has no serotypes, and its molecular evolution is not fully understood. Like FMD, SVA can undergo recombination [18,19,20,21].

For this study, whole-genome sequence data from 47 Canadian samples between 2015 and 2023 collected as part of disease investigations submitted to the Canadian Food Inspection Agency (CFIA), National Centre for Foreign Animal Disease (NCFAD) were obtained by high-throughput sequencing (Supplementary Table S1). All other sequence data (301 sequences) for analysis were obtained from GenBank (Supplementary Table S2). A total of 348 SVA and 3 *Senecavirus cetus* whole-genome sequences were used in phylogenetic and phylogeographic analyses. The molecular evolution and phylogeographic analysis of full genome sequences for all 348 SVA sequences were analyzed over time. In addition, recombination events were analyzed in these sequences.

## 2. Materials and Methods

### 2.1. Sample Preparation

Epithelial tissues, swabs and vesicular fluid were submitted to CFIA/NCFAD for disease investigations to rule out vesicular diseases, including FMD, SVD and VS. All tissue samples for testing were received in universal transport media (UTM) from COPAN Diagnostics. Tissue samples were homogenized in D-PBS to produce 10% weight/volume suspensions. These suspensions were then clarified by centrifugation (2000 g for 20 min at 4 °C) prior to RNA extraction. Swabs (nasal or lesion) were sent in either UTM or dipped in UTM prior to swabbing the animal. Swabs sent without UTM were placed in 1 mL of D-PBS and vortexed to release the virus from the swab. Vesicular fluid was submitted as is or in UTM.

### 2.2. RNA Extraction and Real-Time Reverse Transcriptase Polymerase Chain Reaction (RRT-PCR)

To identify SVA positive samples for further analysis by whole genome sequencing, RRT-PCR was used. RNA extraction from tissue suspensions, swabs and vesicular fluid was performed using the MagMax-96 Viral RNA Isolation Kit, AM1836 (Applied Biosystems, Waltham, MA, USA), following the manufacturer’s protocol. RNA was extracted using a MagMax Express-96 deep well magnetic particle processor (Applied Biosystems) and then amplified by RRT-PCR using previously described primers (SVA 4269- forward primer 5′-TCT CTT GCC CTA ACA CTG GGG—3′ and SVA reverse primer 5′-CTT GCC TCT AAG GAC CAC CACA-3′) and probe [5′ 6FAM- TGG CCC AAA/ZEN†/GTC TCA CCA CTA TGA TCA ATG -IABkFQ†—(3′ ZEN/Iowa Black FQ Black Hole Quencher 1–IDT)] that amplify a 117 bp region of 2C [6]. The template RNA (5 μL) was added to a mastermix comprised of 6.5 μL of 4x TaqMan^®^ Fast Virus 1-Step master mix (Applied Biosystems), 0.2 μM each of the forward and reverse primers and 0.1 μM probe. RRT-PCR was run on either a 7500 Fast (Applied Biosystems) or a QuantStudio 7 Pro (Applied Biosystems) machine using the same cycling conditions: 50 °C for 5 min, 95 °C for 20 s followed by 40 cycles of 95 °C for 15 s and 60 °C for 45 s. A cut-off quantification cycle (Cq) of ≤35.99 was considered positive for the presence of SVA genome in the sample.

### 2.3. Genome Amplification and Whole Genome Sequencing

DNase treatment (DNase I, RNase-Free Life Technologies Catalogue #AM2224) was performed on the extracted RNA, followed by cleanup with an RNeasy MinElute kit (Qiagen, Hilden, Germany). cDNA was prepared using LunaScript (NEB) followed by PCR amplification with NEBNext Ultra II Q5 Master mix using Primal Scheme PCR Tiling primers [22] (Table 1) followed by a cleanup step (Qiagen PCR purification kit) of the combined PCR reactions. The PCR amplicons were used to prepare Illumina Nextera XT libraries following the manufacturer’s protocol. Cleanup of the libraries was performed with Beckman Coulter AMPure XP beads at 0.6×, and then the barcoded libraries were pooled. An Agilent TapeStation 4200 with Agilent High Sensitivity D5000 ScreenTape and High Sensitivity D5000 Reagents (Agilent, Santa Clara, CA, USA) was used to determine the average bp size of the Library Pool. Once the Library Pool has been analyzed on the TapeStation and the concentration has been measured with the Qubit HS DNA kit, the library prep follows the Illumina method to denature the libraries and prepare them to run on the Illumina MiSeq. The resulting fastq files generated by MiSeq were analyzed via the following pipeline.

Preliminary virus detection and assembly on Illumina sequencing data were performed with the CFIA-NCFAD/nf-villumina (v2.0.1) [23] Nextflow [24] pipeline. First, nf-villumina removed Illumina PhiX Sequencing Control V3 reads using BBDuk (v38.96) [25], followed by adaptor removal and quality filtering with fastp [26]. Filtered reads were taxonomically classified with Centrifuge (v1.0.4) [27] and Kraken2 (v2.1.2) [28] using an NCBI nt Centrifuge index built on 4 February 2020, and a Kraken2 index of NCBI RefSeq sequences of archaea, bacteria, viral and the human genome GRCh38 downloaded and built on 7 June 2022. Viral and unclassified reads based on taxonomic classification were retained for de novo assembly with Unicycler (v0.4.8) [29], Shovill (v1.1.0) [30] and MEGAHIT (v1.2.9) [31], and the resulting contigs from each assembly were queried against the NCBI nt database (downloaded 9 January 2020) using blastn (v2.13.0) [32,33] (default parameters except “−evalue 1·e^−6^”). The fastp filtered reads were mapped to the top SVA BLAST match for each sample in Geneious Prime (v2021.2.2, https://geneious.com) (accessed on 27 March 2024) [34] with five iterations of the Geneious assembler on low sensitivity and otherwise default settings. A 75% majority consensus sequence was called with a low coverage threshold of 10× coverage. The resulting consensus sequences were aligned to the de novo assembled contigs with MAFFT (v7.490) [35] to manually check for assembly errors and then checked for the presence of a complete coding sequence for the polyprotein gene in Geneious Prime (v2021.2.2) with the Find ORFs tool.

For Nanopore sequencing, the following method was used: Briefly, the RNA was treated with DNase, followed by cDNA and Primal Scheme PCR in an identical manner as what was prepared for Illumina Sequencing (above). The Nanopore Library Prep was done with the Nanopore Rapid Barcoding Sequencing Kit 96 (Nanopore SQK-RBK110.96) (Oxford Nanopore Technologies, Oxford, UK) following the manufacturer’s protocol. The library was run on the Nanopore GridION using MIN106D spot-on flow cells (flow cell chemistry R9.4.1) (Oxford Nanopore Technologies) with super-accurate base-calling.

For Nanopore sequencing data, the initial analysis was performed by performing adapter trimming with Porechop (v0.2.4) [36] and quality trimming with Chopper (v0.5.0) [37] with a minimum quality score set to 10 and a minimum read length set to 50. All samples were then run through the CFIA-NCAF/nf-virontus (https://github.com/peterk87/nf-virontus, v1.1.0) (accessed on 27 March 2024) [38] Nextflow [24] workflow using the Senecavirus A RefSeq sequence (NC_011349) as a reference and minimum depth of coverage threshold set to 30. An online BLAST [32] search against the NCBI nt database was performed on the resulting consensus sequences, and the workflow was re-run using the top BLAST match with a full-length genome for each sample as a reference. Trimmed reads were mapped to the references in Geneious Prime (v2023.1.1) with the Minimap2 assembler (v2.24) [39] and compared to the previous consensus to manually check the assemblies and produce a 75% majority consensus sequence with a low coverage threshold of 30x coverage. The resulting consensus sequences were checked for a complete coding sequence for the polyprotein gene in Geneious Prime (v2023.1.1) with the Find ORFs tool.

### 2.4. Phylogenetic and Phylogeographic Analysis

The analysis consists of 348 complete and high-quality SVA genomes either sequenced in-house for the Canadian isolates (47) or downloaded from GenBank (accessed on 11 May 2023), including complete SVA genomes from China (*n* = 143), the USA (*n* = 120), Brazil (*n* = 14), previously published genomes from Canada (*n* = 13), Thailand (*n* = 6), Colombia (*n* = 1), India (*n* = 1), Mexico (*n* = 1), Vietnam (*n* = 1) and Chile (*n* = 1). Complete coding sequences were extracted and aligned for all full genome records using MAFFT [v.7.45] [35] under the default settings. Only the sequence records that had collection date information available were included in the final data set. Phylogenetic tree and geographical reconstruction analyses were performed simultaneously using BEAST v1.10.4 [40] software with discrete trait reconstruction under the symmetric substitution model and the Bayesian Stochastic Search Variable Selection procedure enabled. Time-calibrated phylogenetic analysis was performed using a coalescent model with constant population size [41,42] and a general time-reversible substitution model with the across-site rate heterogeneity sampled from a gamma distribution with four discrete categories and relaxed log-normal molecular clock. Four independent analyses were run for 100 million generations each (parameter values recorded every 10,000 generations). To investigate dispersion patterns at different geographical scales, two nested phylogeographical analyses were performed—a coarse phylogeographic reconstruction was performed using all sequence records to infer global-scale patterns, and a finer scale analysis was performed for the sequence records from the USA and Canada only. Stationarity, convergence and the burn-in fraction across independent runs were assessed in Tracer v1.7 [43]. The tree and log files were then combined using the LogCombiner module of the BEAST software, and the final maximum clade credibility tree was produced using TreeAnnotator [40]. Phylogeographic reconstruction and visualization were performed using SpreaD3 [44]. The final phylogenetic trees were visualized with FigTree v.1.4.4 (https://github.com/rambaut/figtree) (accessed on 27 March 2024) and Inkscape (https://inkscape.org/) (accessed on 27 March 2024).

### 2.5. Recombination Analysis

Intra-specific recombination events have been reported in Senecavirus A [45]. To identify potential genome recombinations in our expanded Senecavirus A dataset, a series of recombination analyses were performed using the RDP5 v.5.30 software [46] under the default settings and with the *p*-value cut-off set to 0.01. Initial identification of potential recombination events and breakpoints was performed using five detection methods (RDP, GENECONV, 3Seq, MaxChi, Chimaera). The identified recombination events were then checked with BootScan and SiScan. Recombination events detected by at least five out of seven methods were selected as plausible.

### 2.6. Selection Analysis

To identify protein sites experiencing selection pressure within each gene of the SVA genome, we used the fast unconstrained Bayesian approximation (FUBAR) and single-likelihood ancestor counting (SLAC) methods [47]. Selection inference analysis was performed using nucleotide sequence alignments of each major gene in the global SVA dataset (*n* = 348 sequences). FUBAR and SLAC analysis for the global dataset were performed using HyPhy v.2.2.4 [48,49]. Protein sites were inferred to be under selection if the posterior probability of inferred selection pressure (positive or negative) was ≥0.9 (FUBAR) or with a *p*-value < 0.1 (SLAC). Only the sites identified as being under selection by both methods were selected for the final result interpretation.

## 3. Results

### 3.1. Phylogenetic Analysis of SVA Genome Sequences

**Figure 1:**
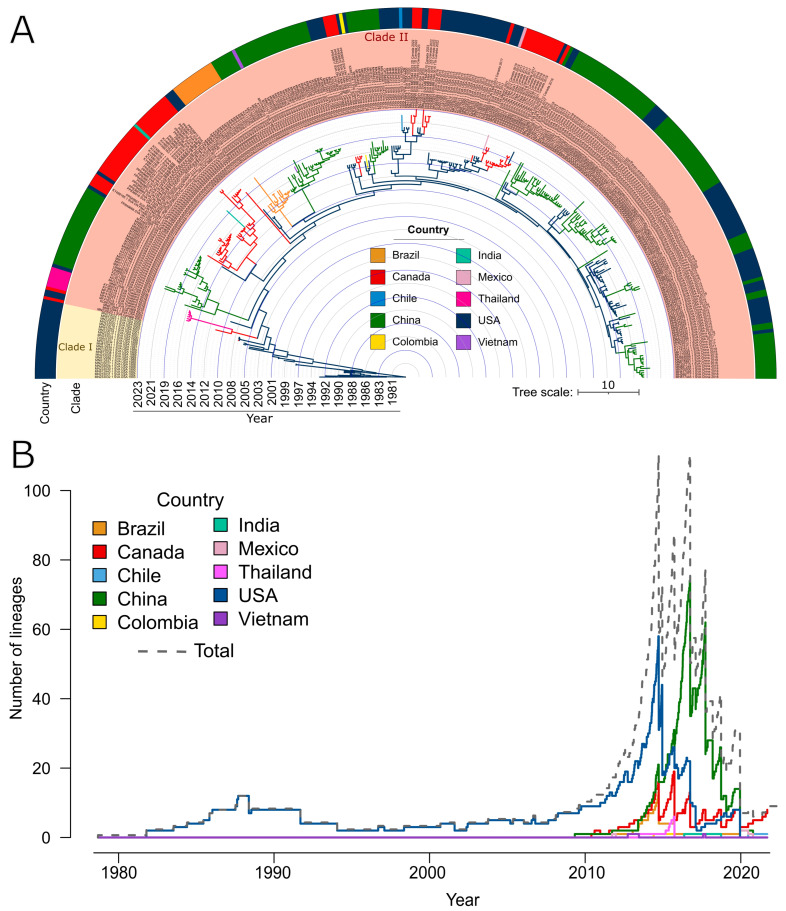
Time-calibrated phylogeographic analysis of SVA using coalescent model and Bayesian Stochastic Search Variable Selection procedure. (**A**) Phylogenetic tree with internal branches coloured according to the inferred country of origin. The time scale is shown in years. Clades are highlighted and labelled following work by Wu et al. [52]. (**B**) Number of SVA lineages through time by country. The dashed grey line represents the total number of lineages.

In this study, whole genome sequences were obtained either from positive submission samples (*n* = 47; Supplementary Table S1) or from GenBank (*n* = 301; Supplementary Table S2). The sequences were analyzed both phylogenetically and geographically over time. Overall, the topology of the phylogenetic tree from this study is consistent with other published phylogenies with fewer sequences (Figure 1) [50]. The sequences can be separated into two clades (pre- and post-2007), as defined by Wu et al. [51]. Other publications have split the data into multiple clades but have only referenced South China isolates and based their classification on percent sequence identity [52].

The median age of the root of the phylogenetic tree was estimated to be December 1978 (95% highest posterior density interval (HPDI) was between April 1972 and August 1983), which suggests that SVA could have emerged nearly a decade before it was first identified and sequenced. Clade I includes all pre-2007 sequence records that represent the earliest known genetic lineages of SVA. Clade II is the largest of the two groups. It includes all contemporary strains of SVA and comprises several genetically distinct lineages that, in most cases, represent monophyletic groups that emerged as a result of incursion/phylogeographic spread events followed by diversification. The relaxed molecular clock analysis revealed that the SVA evolutionary rate was steady across lineages and time with an average substitution rate of 3.92 × 10^−3^ substitutions/site/year (95% HPDI 3.24 × 10^−3^; 4.60 × 10^−3^).

### 3.2. SVA Inferred Spread from the United States to Canada

**Figure 2:**
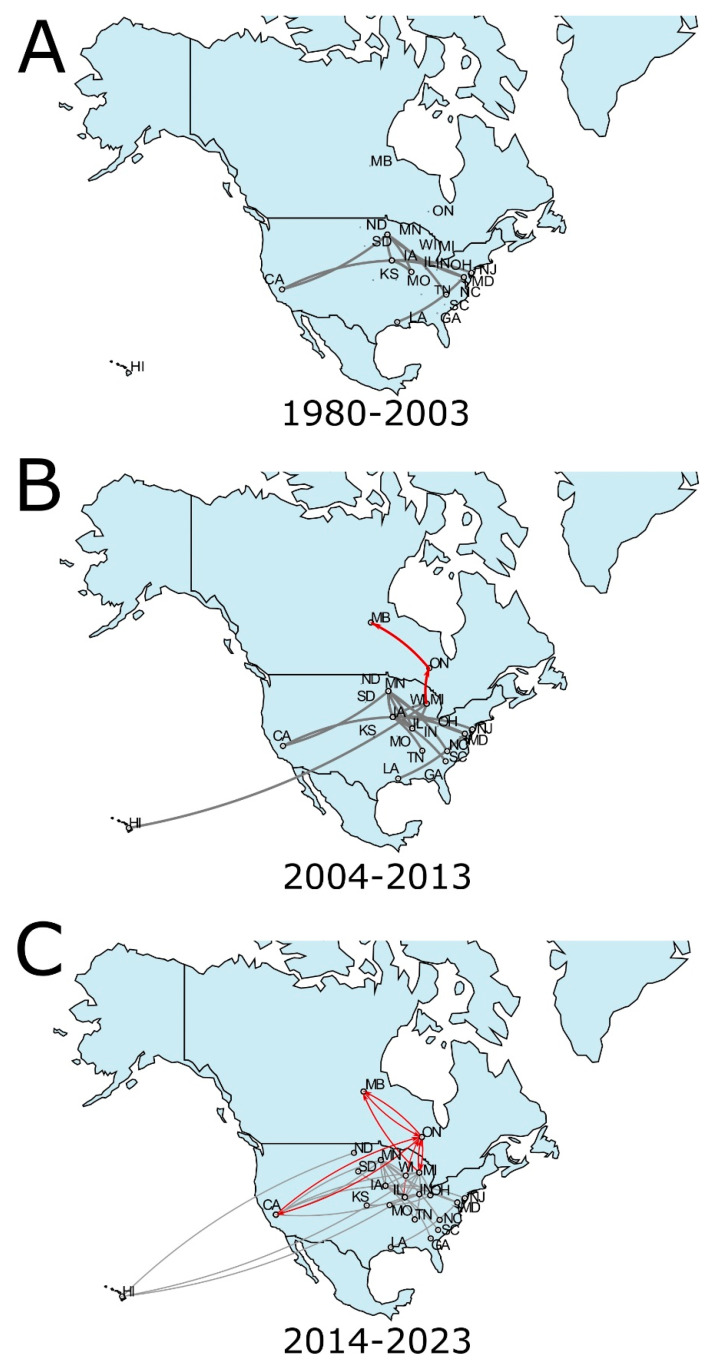
Inferred paths of geographic spread of SVA in North America. (**A**) Detection and initial spread within the USA, 1980–2003; (**B**) first incursion across-border transmission into Canada and more expansive circulation within the USA, 2004–2013; (**C**) spread of SVA within the USA and multiple transmissions into and within Canada, 2014–2023. Dispersal pathways within Canada and across the USA border are shown in red. Pathway analysis was performed using a phylogeographic diffusion model in discrete space implemented in BEAST 1.10.4.

Based on the phylogeographic data in this study, the United States was identified as the centre of origin (Figure 1 and Figure 2A). Following the emergence of SVA in the USA, with regional circulation within the country and suspected cross-border spread into Canada as early as 2003, and was first detected in Canadian swine in 2007 (Figure 2B). The post-2007 period is marked by a substantial increase in SVA lineages (Figure 1B). Between 2007 and 2016, the USA had the highest number of SVA lineages with extensive circulation within the country and cross-border spread into Canada, which similarly experienced an increase in the number of circulating lineages of SVA during that time (Figure 1B and Figure 2). Based on the phylogeographic reconstruction of existing data, Iowa was the most likely centre of origin of SVA within the US, with the subsequent spread to neighbouring Minnesota and Illinois. By 1997, the virus had spread to the mid- and south Atlantic USA (Maryland, New Jersey and North Carolina) and had reached Louisiana in the South. During the next decade (1997–2007), SVA spread to the Pacific coast of the USA in the transmission event from Minnesota to California. The virus circulated in the Midwest, spreading from Minnesota to Illinois and from Iowa farther southeast, reaching Tennessee and South Carolina. Iowa was also inferred to be the source of SVA spread to Michigan. Subsequently, Michigan was inferred as the point of origin for the cross-border incursion of SVA into Canada (Ontario). Within Canada, SVA spread from Ontario to Manitoba as early as 2011. In the USA, the Midwest was the region of the most active inter-state transmissions of SVA and a source region for long-distance spreads such as from Michigan and Illinois to Hawaii. The 2013–2023 decade was marked by the rapid increase in spread events and the expansion of the transmission networks in the USA and Canada (Figure 2C). During that time, incursions of SVA from the USA into Canada were inferred from Illinois, Michigan and California. Simultaneously, SVA was circulating within Canada and spread from Manitoba to Ontario. This observation indicates that SVA has spread back and forth between Canada and the United States multiple times.

### 3.3. SVA Inferred Spread from North America to Many Other Countries in the World

**Figure 3:**
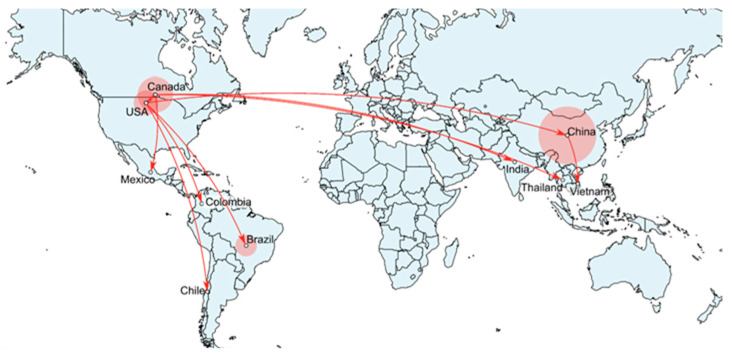
Inferred paths of the international spread of SVA between 1988 and 2023 based on the phylogeographic analysis using 348 complete genome records.

Current evidence suggests that the United States, specifically Iowa (Figure 3), was the centre of origin and diversification of SVA for nearly three decades since its emergence. The subsequent inferred spread to other countries largely followed the patterns of trade routes, with Canada and China being the first countries where SVA was detected. Phylogeographic patterns indicate that both China and Canada have SVA lineages from several distinct incursion events. Each independent spread event led to SVA diversifying and forming distinct monophyletic groups. In China, at least eight distinct phylogenetic clusters can be identified (Figure 1A). The rapid spread and diversification of SVA in China in 2014–2020 resulted in the country’s highest number of SVA lineages surpassing that of the United States (Figure 1B). In contrast, incursion into Brazil appears to be a single event that may have occurred in early 2015 and gave rise to all Brazilian sequences included in the data set. In two instances, it also appears that the virus spread from Canada back to the United States (2016, 2020). The movement of pigs between Canada and the United States follows import/export regulations. Feral swine infected with SVA could be a possible transmission route [53]. The SVA samples from Thailand were most closely related to the SVA samples from Canada. The Canada to Thailand dispersal route can be inferred as occurring sometime in 2016. The SVA sample from India is deeply nested within a clade of Canadian SVA sequences, and the Canada to India pathway of dispersal in 2019 was the most probable. Similarly, the spread of SVA was inferred from the United States to Colombia (2016), China to Vietnam (2018) and Canada to Mexico (2021). The recent report of SVA from Chile [12] was inferred to result from a spread event from the United States in 2022.

### 3.4. SVA Has Undergone Recombination in Canada, the United States and China

**Figure 4:**
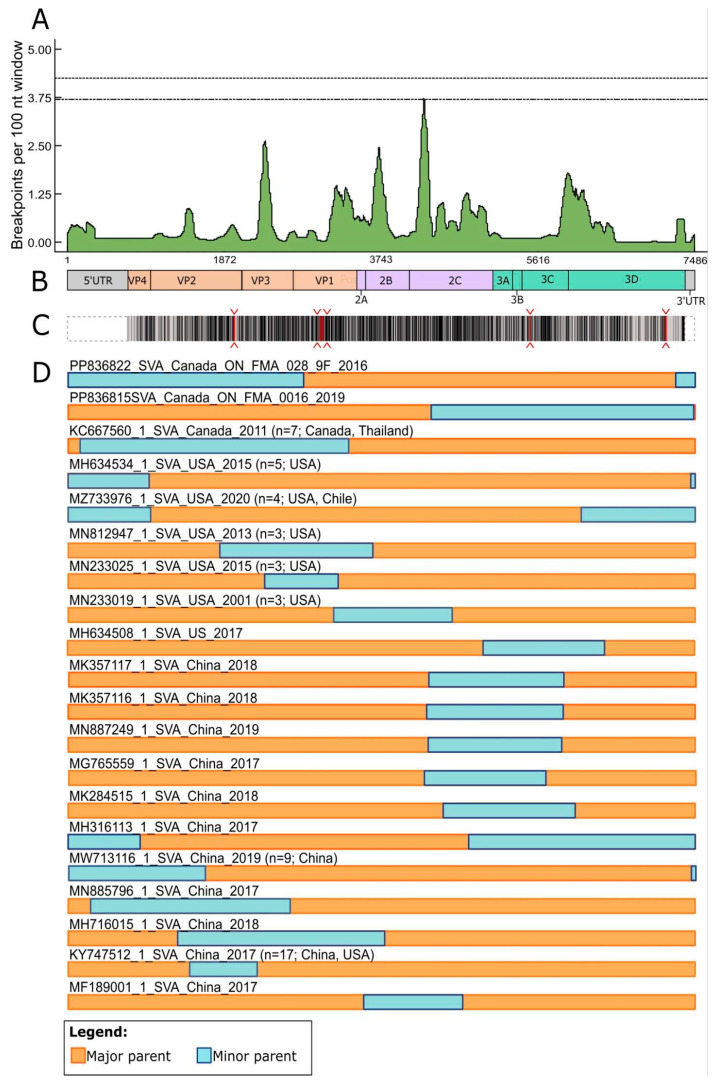
Patterns of recombination events and selection in SVA genomes. (**A**) Distribution of recombination breakpoints across the genome based on the inferred recombination events. The number of breakpoints was calculated using a sliding window approach with a window size of 100 nt. (**B**) Schematic of the SVA genome organization. (**C**) Patterns of the selection pressure on protein sites across the SVA genome. Black lines represent protein sites inferred to be under negative selection (*n* = 1159); red lines and carets indicate sites inferred to be under positive (diversifying) selection (*n* = 5); no selective pressure was inferred for the sites shown in light grey (*n* = 1017). (**D**) Patterns of recombination inferred from complete-genome records (*n* = 348 sequences); orange colour represents portions of the genome from a major parental sequence; blue colour represents the portion of the genome from minor parental sequences. Detailed information about each identified recombination event is provided in Table 1. Numbers and countries in parenthesis indicate the number and provenance of sequence records in which a recombination pattern was inferred (if more than one recombinant sequence was identified for a recombination pattern).

SVA genomes were assessed for the occurrence of recombination. As it is characteristic of picornaviruses [54], including Senecavirus A [55], recombination breakpoints have been identified at multiple points across the genome. Potential recombination hotspots were identified in the VP1, VP3, 2B, 2C and 3D regions of the genomes, as indicated by the high number of inferred breakpoints (Figure 4A). Recombination was observed in SVA genome sequences from Canada, the United States and China (Figure 4D). The location of recombination sites was variable and occurred at different genome regions. Among the three identified recombinant sequences from Canada, two had a similar recombination pattern with a breakpoint at the end of the P1 region, with the majority of that region (VP4, VP2, VP3 and partial VP1 genes) coming from a minor parent and the rest of the genome (P2 and P3 regions) contributed by the major parental strain. The same pattern of recombination (P1 from the minor parent and P2 + P3 from the major parent) was also identified in the six sequence records reported from Thailand (Supplementary Table S1), which supported the inferred Canada-to-Thailand transmission pathway (Figure 3). The third recombinant strain from Canada had a distinct pattern with a crossover between the parental genomes within the 2C gene (P2 region). Among the recombinant strains from the USA and China, the most common patterns of recombination included the following: (1) crossover within the P1 region with most of P1 and complete P2 and P3 contributed by the major parent; (2) crossover at the breakpoints in the P1 and P3 regions and (3) crossover at the breakpoints in the P2 and P3 regions. The recombination pattern involving crossover in the P1 and P3 regions was identified in three isolates from the USA collected in 2020 and a more recent sample reported from Chile in 2022 (Figure 4D, Supplementary Table S3). This suggests that the recombinant strains that emerged in the USA successfully persisted and spread to South America.

Despite the inferred relatively high frequency of the recombination breakpoints and the variety of recombination patterns, selection analysis revealed that the majority of the polyprotein sites are under negative selection, which limits genomic diversity to preserve the proper function of the proteins and removes potential deleterious mutations (Figure 4C). Slightly over 53% of the amino acid sites were inferred to be under negative selection, while only five sites (~0.2%) were inferred to be under diversifying (positive) selection. Of the five sites inferred to be under positive selection, three are located in the structural VP1 (*n* = 2) and VP2 (*n* = 1) genes that play an important role in capsid formation and receptor recognition. The remaining two positively selected sites were inferred in the non-structural P3 regions within the protease (3C) and RdRp (3D) genes (Figure 4C).

### 3.5. Placement of SVA Within the Picornavirus Subfamily Capthovirinae

**Figure 5:**
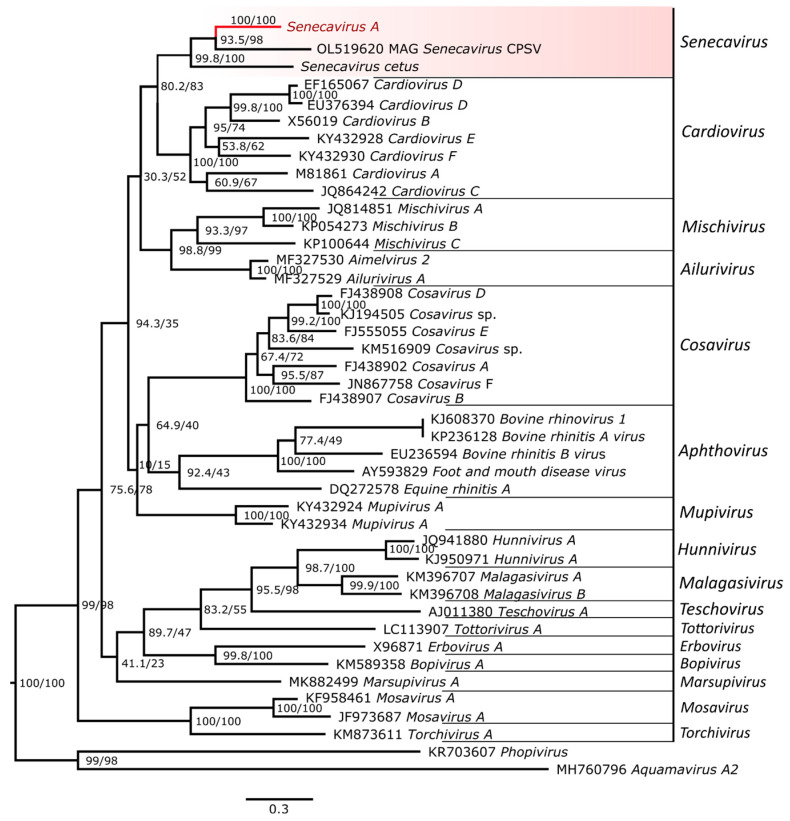
Maximum likelihood phylogenetic tree showing placement of Senecavirus A within the picornavirus subfamily Capthovirinae. Phylogenetic tree was inferred based on the nucleotide sequences of the conserved 3Dpol gene. Individual sequences of Senecavirus A used in the analysis formed a strongly supported monophyletic group and were collapsed in the tree to represent a single lineage of the species. Genus *Senecavirus*, which includes a partial *Senecavirus*-like genome identified in pangolin, OL519620 MAG Senecavirus CPSV and *Senecavirus cetus* isolated from cetaceans, is highlighted in red. Phylogenetic analysis was performed using IQ-Tree web server 1.6.12. The trees were rooted to the outgroups, Phopivirus and Aquamavirus A2, members of the subfamilies Heptrevirinae and Paavivirinae, respectively. Scale bar represents estimated average number of substitutions per site.

A new species in the Senecavirus genus, Senecavirus cetus, was recently reported from a harbour porpoise and beluga whale found dead and stranded in Alaska, USA, in 2017 and 2019, respectively [56]. This new virus, along with a previously reported partial genome of a Seneca-like virus isolated from pangolin, expands the previously monospecific genus Senecavirus to three distinct lineages (Figure 5; Supplementary Table S4). The discovery of a senecavirus in marine mammals with 58.3% genome-wide pairwise nucleotide identity to SVA in swine suggests an interesting but poorly understood part of senecavirus evolution that remains to be elucidated.

## 4. Discussion

The phylogenetic analysis reported here demonstrated that SVA can be assigned into two main groups (Figure 1), clade I (pre-2007 strains) and clade II (post-2007 strains), and is in agreement with a previous report [51]. Further assignment of additional clades from clade II is possible based on the genetic divergence of lineages that form distinct monophyletic groups [57]. The genetic structure of the SVA phylogeny is greatly influenced and directed by the spread events that lead to the formation and diversification of distinct phylogenetic clades. Signatures of the ancestral genetic diversity and recombination patterns can provide insights into the phylogenetic origin and geographic pathways of transmission of viruses, as has been shown for picornaviruses [58,59,60] and many other human and animal viruses [61,62,63,64,65]. In this study, inferred patterns of recombination (Figure 4D) were overall consistent with the phylogeographic reconstruction (Figure 1A). The same pattern of genome recombination was found in SVA strains from Canada and Thailand, which were also inferred to be sister lineages in the phylogenetic tree; similarly, strains from the USA and Chile that shared the same inferred pattern of recombination were most closely related to each other in the inferred phylogeny. However, interpretation of the recombination patterns should be taken with caution as recombination events can mislead phylogenetic reconstructions and can introduce additional uncertainty in the results when recombination donor sequences cannot be identified [65,66]. Since recombination in SVA occurred in different regions of the genome, a partial sequence would not be able to detect all the occurrences of recombination; therefore, whole genome sequencing is required.

SVA, like other RNA viruses, exhibits high mutation rates [67,68]. Our analysis estimated an average substitution rate of 3.92 × 10^−3^ substitutions/site/year, which is consistent with the previously reported SVA evolutionary rates by Zeng et al. [50]. High mutation rates generate a large number of genetic variants, which are subject to selection pressures. In the SVA genome, over 50% of the polyprotein sites were inferred to be under purifying selection, which removes detrimental mutations and ensures the proper function of the polyprotein components. Similarly, a high proportion of protein sites under negative selection have been reported in other picornaviruses, such as *Enterovirus* and *human rhinoviruses* [69,70]. Positive selection that promotes genetic diversification was only detected at five sites within the VP1, VP2, 3C and 3D genes. These proteins play an important role in receptor recognition and binding, evasion of host immune response and replication [71]. Therefore, diversifying selection in these regions of the SVA genome could promote the emergence of strains with higher infectivity.

Existing sequence data suggest that SVA originated in Iowa, in the Midwest United States, which is the largest pork-producing state in the United States [72]. Clade 1 viruses are likely extinct as they are no longer detected in the field. Clade II SVA first inferred spread from the United States to Canada in 2007. The cross-border spread of SVA between the United States and Canada has occurred several times since. This is expected due to the highly integrated swine industries in North America. The phylogeographic data suggest inferred SVA spread from the United States to Brazil, Columbia, Chile and China on 10 separate occasions between 2008 and 2019. In addition, SVA spread was inferred from China to Vietnam. Furthermore, the results suggest inferred SVA spread from Canada to Thailand, India and Mexico. A limitation of this study is that the accuracy of phylogeographic analysis can be impacted by many factors. One of the most important factors is the migration rate of the virus, where accuracy is reduced with higher migration rates [73]. SVA migration rates are relatively high, according to Zeng et al. [50], being a limitation of the analysis. The new sequence data and analysis presented in this paper describe the spread of SVA in more detail compared to a previous study from Gao et al., which characterizes the inferred spread of SVA from the United States to the rest of the world [57] and builds on the sequence data from Chinese isolates [52]. The data from this paper disagree with Wu et al. [51], which described the spread of SVA from Brazil to the USA and China, using Bayesian phylogeographic analysis. Wu et al. [51] only looked at the origin of the SVA outbreaks since 2015 and did not incorporate earlier sequence records that come predominantly from the USA, Canada and China.

One limitation of the existing phylogeographic data is there remain gaps since sequence data are predominantly from the USA, Canada and China. These knowledge gaps can be problematic as it is also possible that SVA spread from the United States, and not Canada, into Mexico. The transmission of SVA can occur through the trade and transport of infected swine and through either contact or the fecal–oral–nasal route. It is unlikely that airborne transmission can occur with SVA as with foot-and-mouth disease (FMD). Legal exports of pigs and their products from China to countries free of FMD and African swine fever (ASF) are restricted due to trade restrictions resulting from the presence of FMD and ASF in China. It is unlikely that SVA would spread back to North America through pigs and pork products. However, it is possible that SVA can spread through contaminated feed ingredients [74]. SVA viability in feed and spiked swine bone marrow tissue has been estimated to last up to 118 days, indicating that SVA is stable in these situations [75]. Recently, it has been demonstrated that SVA was associated with feed imports from endemically infected countries; however, the names of countries and companies associated with the infected feed were not disclosed [76].

In Canada, SVA-caused clinical disease has only been confirmed in the provinces of MB and ON. The reason for this is unknown, as pigs and their products move throughout Canada. In MB, SVA is routinely isolated from an assembly yard, where animals are mixed, and increased stress is likely promoting the vesicular lesions caused by SVA.

## 5. Conclusions

SVA continues to evolve through high mutation rates as well as through recombination. The inconsistency in the development of clinical signs of SVA makes passive surveillance limited use for SVA since many infections are missed. Since SVA does not affect trade, the reporting of SVA is limited to clinical cases of disease in Canada. The spread of SVA into several new regions like Asia, South America and Europe has occurred, and it is likely that SVA will continue to spread worldwide since it is a disease that does not affect trade.

## Figures and Tables

**Table 1:** Representative parental sequences and recombination breakpoint positions of the recombination events shown in Figure 4.

	Recombinant Sequence	Breakpoint Position in Recombinant Sequence		Representative Major Parental Sequence	Representative Minor Parental Sequence
		Begin	End		
1	PP836822_SVA_Canada_ON_FMA_028_9F_2016	7107	2700	PP836819_SVA_Canada_ON_FMA_025_2C_2016	PP836823_SVA_Canada_ON_FMA_029_2D_2016
2	PP836815_SVA_Canada_ON_FMA_0016_2019	4200	7228	KY486164_1_SVA_MB_NCFAD_119_7_Canada_2015	MZ733980_1_SVA_USA_2020
3	KC667560_1_SVA_Canada_2011	95	3264	MN233033_1_SVA_USA_1999	KY486157_1_SVA_MB_NCFAD_104_6_Canada_2015
4	MH634534_1_SVA_US_2015	7267	883	MK357115_1_SVA_China_2018	Unknown (MK357117_1_SVA_China_2018)
5	MZ733976_1_SVA_USA_2020	5994	884	PP836804_SVA_Canada_MB_FMA_0015_122_2022	Unknown (SVA_Canada_ON_FMA_0011_2019)
6	MN812947_1_SVA_USA_2013	1683	3527	MN812946_1_SVA_USA_2009	KY486158_1_SVA_MB_NCFAD_104_9_Canada_2015
7	MN233025_1_SVA_USA_2015	2242	3144	MH634525_1_SVA_US_2015	Unknown (MN700930_1_SVA_China_2018)
8	MN233019_1_SVA_USA_2001	3067	4498	MN812941_1_SVA_USA_1997	MN812943_1_SVA_USA_2006
9	MH634508_1_SVA_US_2017	4860	6317	MH634506_1_SVA_US_2017	MZ733976_1_SVA_USA_2020
10	MK357117_1_SVA_China_2018	4199	5819	MN781983_1_SVA_China_2018	MK357115_1_SVA_China_2018
11	MK357116_1_SVA_China_2018	4181	5819	ON868370_1_SVA_China_2020	MK357115_1_SVA_China_2018
12	MN887249_1_SVA_China_2019	4173	5788	MW117127_1_SVA_China_2020	MK802891_1_SVA_China_2018
13	MG765559_1_SVA_China_2017	4139	5612	MG765560_1_SVA_China_2017	MG765550_1_SVA_China_2017
14	MK284515_1_SVA_China_2018	4355	5944	MG765556_1_SVA_China_2017	MT840202_1_SVA_China_2018
15	MH316113_1_SVA_China_2017	4678	789	ON868376_1_SVA_China_2021	MG765556_1_SVA_China_2017
16	MW713116_1_SVA_China_2019	7284	1559	KX751946_1_SVA_China_2016	KX173340_1_SVA_China_2015
17	MN885796_1_SVA_China_2017	222	2580	Unknown (KU954090_1_SVA_USA_2015)	MK284514_1_SVA_China_2018
18	MH716015_1_SVA_China_2018	1211	3693	MH634515_1_SVA_US_2017	Unknown (OP184809_1_SVA_USA_2013)
19	KY747512_1_SVA_China_2017	1367	2186	KU954090_1_SVA_USA_2015	MG765557_1_SVA_China_2017
20	MF189001_1_SVA_China_2017	3436	4628	MF189000_1_SVA_China_2017	KY747511_1_SVA_China_2017

## Data Availability

The data presented in this study are openly available in Genbank (Supplementary Table S2).

## Supplementary Materials

The following supporting information can be downloaded at: https://www.mdpi.com/article/10.3390/v17020141/s1, Supplementary Table S1: GenBank accession numbers and genome completeness of the 47 SVA sequence records reported in the study. Supplementary Table S2: Accession numbers of the sequence records used in phylogeographic analysis. Supplemental Table S3: Recombination breakpoint positions. Supplemental Table S4: Accession numbers of the sequence records used to reconstruct phylogenetic tree of the subfamily Capthovirinae.
